# First Occurrence of *Wronascolex* sp. (Palaeoscolecida, Priapulida) in the Cambrian Tianpeng Formation (Wuliuan Stage), Southeastern Yunnan, South China: Implications for a New Burgess Shale-Type Lagerstätte

**DOI:** 10.3390/life16040640

**Published:** 2026-04-10

**Authors:** Shang-Yun-Zhi Xiao, Liu-Run-Xuan Chen, Shi-Tao Zhang, Dai Zhang, Xian-Chao Chen, Yu-Kai Hu, Qiu-Yun Song, Xiao-Qi Yang, Ruo-Han Zuo, Guang-Xu Zhang

**Affiliations:** 1 Faculty of Land Resources Engineering, Kunming University of Science and Technology, Kunming 650093, China; 2 School of Geosciences, Yunnan University, Kunming 650500, China; 3 Key Laboratory of Critical Minerals Metallogeny in Universities of Yunnan Province, School of Earth Sciences, Yunnan University, Kunming 650500, China; 4 Kunming Prospecting Design Institute of China Nonferrous Metals Industry, Kunming 650051, China; 5 Yunnan Provincial Key Laboratory of Geotechnical Engineering and Geological Hazards, Kunming 650051, China; 6 Analytic & Testing Research Center of Yunnan, Kunming 650093, China; 7 Research Center for Analysis and Measurement Kunming, University of Science and Technology, Kunming 650093, China; 8 College of Resources and Environmental Engineering, Key Laboratory of Karst Georesources and Environment, Ministry of Education, Guizhou University, Guiyang 550025, China; 9 Guizhou Provincial Key Laboratory for Palaeontology and Palaeoenvironment, Guiyang 550025, China

**Keywords:** *Wronascolex*, Cambrian (Wuliuan), Southeast Yunnan Province, South China, Burgess Shale-Type Lagerstätte

## Abstract

We report the discovery of a new palaeoscolecid worm specimen from the Bainiuchang area, southeastern Yunnan, China. The specimen exhibits a cylindrical body with annulations, each bearing two rows of *Hadimopanella*-type sclerites, along with plates, platelets, microplates, and implanted plates. These features are compatible with the diagnosis of the genus *Wronascolex*, and the specimen is tentatively assigned to *Wronascolex* sp. However, given the limited number and preservation of the available specimens, which preclude a detailed demonstration of the scleritome morphology for comparison with other palaeoscolecid worms, this assignment should be treated as tentative. This specimen may be the first record of a soft-bodied fossil from the Miaolingian Series (Wuliuan Stage) strata of southeastern Yunnan. Its taphonomic features—preservation as carbonaceous compressions accompanied by iron-rich films—are broadly consistent with Burgess Shale-type (BST) preservation. Whole-rock geochemical analysis of samples from the fossil-bearing interval yielded redox proxy values suggestive of suboxic to weakly reducing depositional conditions, broadly comparable to those reported from some BST deposits, such as the Mackenzie Mountains locality of Canada. However, these geochemical results are preliminary and based on a limited number of samples. Taken together, these observations suggest the possibility that the Bainiuchang area may host a BST Lagerstätte. Should this be confirmed, such a deposit would postdate the Chengjiang and Guanshan biotas (Cambrian Series 2, eastern Yunnan) and predate the Fulu biota, which is the only confirmed BST Lagerstätte in southeastern Yunnan to date. Furthermore, this discovery extends the known paleogeographic range of the genus *Wronascolex* southward to the southwestern margin of the South China Block. It also represents, to our knowledge, the first reported occurrence of soft-bodied fossil preservation in the Wuliuan Stage of Yunnan Province.

## 1. Introduction

Palaeoscolecids are an extinct group of ecdysozoan worms that were widely distributed in marine benthic communities from the early Cambrian to the late Silurian (Ludfordian). They are characterized by an elongated, cylindrical body bearing an annulated trunk ornamented with tessellating organo-phosphatic plates arranged in circular patterns, a layered cuticle, and an armored introvert [1]. Disarticulated phosphatic sclerites of palaeoscolecids, widely recovered as small shelly fossils (SSFs), are formally classified into four sclerite-based parataxa, *Hadimopanella* Gedik, 1977 [2], *Milaculum* Müller, 1973 [3], *Utahphospha* Müller and Miller, 1976 [4], and *Kaimenella* Märss, 1988 [5], differentiated by specific external ornamentation patterns [6,7,8]. These parataxonomic genera were originally described from isolated elements of uncertain biological affinity but were subsequently recognized as dermal sclerites of palaeoscolecid worms [8,9]. Studies of articulated trunk fragments and Orsten-type cuticle arrays have further elucidated the scleritome structure, enabling reconciliation of sclerite-based taxonomies based on isolated sclerites with whole-body taxonomies based on complete or semi-complete specimens [10,11]. The morphology, arrangement, and size differentiation of plates, platelets, and microplates within the scleritome currently serve as the primary framework for genus-level classification [11]. Among the whole-body genera, *Palaeoscolex* Whittard, 1953 [6] is now restricted to Ordovician forms characterized by *Milaculum*-type sclerites, while *Wronascolex* Ivantsov and Zhuravlev, 2005 [12] encompasses Cambrian macroscopic palaeoscolecids bearing *Hadimopanella*-type sclerites with 3–10 nodes arranged in a single circle [12]. Other notable genera include *Tabelliscolex* Han et al., 2007 [7] and *Mafangscolex* Luo et al., 2014 [13]. All of these genera have been documented from Yunnan Province, South China [1,7,13,14,15,16,17], underscoring the region’s significance for understanding palaeoscolecid diversity.

Palaeoscolecid fossils are known from four principal preservational modes: (1) isolated phosphatic sclerites recovered as small shelly fossils (SSFs) [17]; (2) phosphatised cuticle fragments preserved three-dimensionally in Orsten-type assemblages [18]; (3) organic-walled microfossils called small carbonaceous fossils (SCFs) [19]; and (4) macroscopic compressions in Burgess Shale-type (BST) deposits. In addition, palaeoscolecids have been linked to certain ichnofossils, although the trace-fossil record is discussed separately below because it does not represent a body-fossil preservational mode per se. The first three modes—SSFs, Orsten-type cuticle fragments, and SCFs—are by far the most commonly encountered, but they typically yield only isolated plates or incomplete trunk fragments that lack whole-body information [5]. Consequently, a substantial proportion of described palaeoscolecid taxa are based on limited and often non-overlapping morphological data, which complicates cross-comparisons between species described from different preservational modes [11,14].

Palaeoscolecids have been hypothesized to have been infaunal burrowers and potential producers of certain ichnofossils, supported in some cases by the co-occurrence of body and trace fossils [20]. However, the life habit of palaeoscolecids remains debated, as body fossils frequently occur in horizons lacking associated burrows [21]. Moreover, the absence of body fossils in most ichnofossil records severely limits the taxonomic utility of trace fossils for this group.

In contrast, BST fossils are macroscopic compressions that preserve soft tissues as primary carbonaceous remains, sometimes supplemented by secondary mineralization such as aluminosilicification or pyritization, typically within fine-grained marine mudstones [22,23]. Crucially, BST deposits provide the only preservational window in which complete or near-complete palaeoscolecid body plans—including the introvert, gut, and posterior structures—can be observed [11,24]. BST deposits are known from multiple palaeocontinents, with over 40 Cambrian sites documented globally [23]. Among these, the Chengjiang, Guanshan, and Kaili biotas of South China [15,16,25,26] are particularly significant for palaeoscolecid research, as they have yielded exceptionally well-preserved macroscopic specimens that enable the study of both scleritome architecture and internal soft anatomy. Palaeoscolecids are a recurring, if relatively uncommon, component of many BST assemblages [24,27], and the exceptional preservation afforded by these deposits has been instrumental in elucidating their body plan and scleritome organization.

Cambrian macroscopic palaeoscolecids preserved in BST deposits have traditionally been assigned to *Palaeoscolex* Whittard, 1953 [6,12]. However, a major taxonomic revision by García-Bellido et al. (2013) [11] demonstrated that many of these Cambrian species had been misassigned. Because palaeoscolecid compressions generally exhibit similar elongated cylindrical forms with fine annulations, differentiation at the genus and species levels requires detailed SEM examination of sclerite morphology, which is frequently unavailable in older collections [1,11]. García-Bellido et al. (2013) [11] proposed that the genus *Wronascolex* Ivantsov and Zhuravlev, 2005 [9] should encompass all macroscopic palaeoscolecids whose annulations bear one to four rows of *Hadimopanella*-type circular phosphatic sclerites, each with an ornamented upper surface consisting of a single circle of four to ten nodes and an occasional central node. Under this framework, several Cambrian species formerly assigned to *Palaeoscolex* were transferred to *Wronascolex*, including *W. antiquus* (Glaessner, 1979) [28,29] from the Emu Bay Shale of South Australia and *W. ratcliffei* (Robison, 1969) [21,30] from the Spence Shale of Utah. It should be noted, however, that the generic assignment of *W. ratcliffei* has since been questioned by Whitaker et al. (2020) [27], who erected the new genus *Utahscolex* for this species based on SEM-EDS data revealing the absence of node ornamentation and of platelets and microplates—features considered diagnostic for *Wronascolex*. This ongoing revision underscores the challenges inherent in palaeoscolecid systematics. Conversely, *Palaeoscolex* sensu stricto is now restricted to Ordovician species bearing Milaculum-type sclerites, with *P. piscatorum* Whittard, 1953 as the type species [6,12,31]. The present study follows the taxonomic framework of García-Bellido et al. (2013) [11], in which all Cambrian macroscopic palaeoscolecids bearing *Hadimopanella*-type sclerites are assigned to *Wronascolex*.

Since that revision, *Wronascolex* has become a fairly diverse and cosmopolitan genus, with species documented from Cambrian Stage 4 to the Drumian across South China, Siberia, Australia, Spain, and Utah [1,27]. Moreover, the recent discovery of *W. superstes* from the Late Ordovician Tafilalt Lagerstätte of Morocco has extended the total range of the genus to over 60 million years [32]. Nevertheless, the diversity of sclerite patterns and the fragmentary nature of many specimens mean that the taxonomy and biostratigraphic utility of *Wronascolex* remain problematic [1].

Here, we report a new palaeoscolecid specimen, tentatively assigned to *Wronascolex* sp., from the Wuliuan Stage (Miaolingian Series) Tianpeng Formation in the Bainiuchang area, southeastern Yunnan Province, South China. The aims of this study are threefold: (1) to describe the morphology and scleritome of this specimen and evaluate its generic assignment; (2) to characterize its taphonomic features and assess their comparability with Burgess Shale-type preservation; and (3) to present preliminary whole-rock geochemical data from the fossil-bearing horizon as a first-order approximation of the depositional redox conditions. Taken together, these observations are intended to provide an initial assessment of whether the Bainiuchang area has the potential to yield a BST-type fossil assemblage—a hypothesis that will require testing through future, more extensive fieldwork and analytical programs.

## 2. Geological Setting

**Figure 1:**
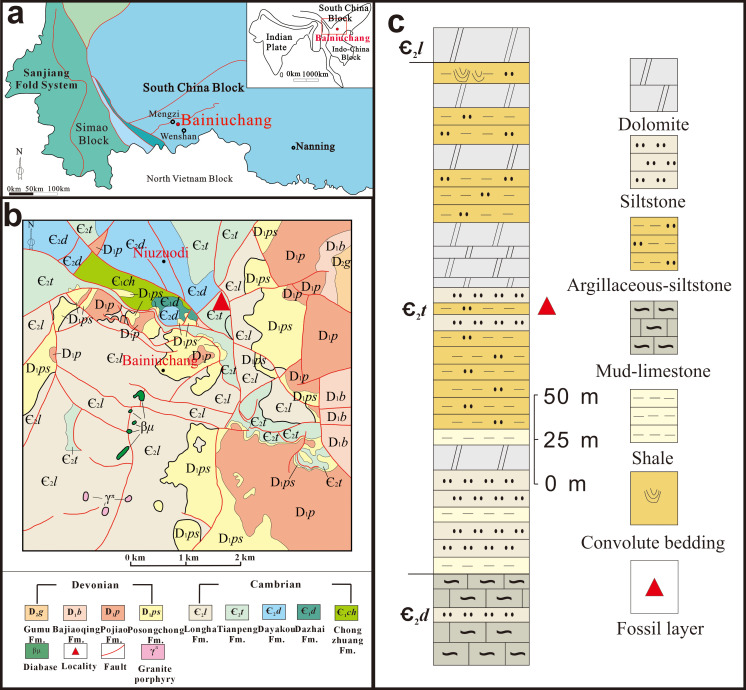
(**a**) Geotectonic location map of South China. (**b**) Geotectonic location map of southeastern Yunnan. (**c**) Lithological column of the Niuzuodi–Feigucun section, Bainiuchang area (pictures are modified according to [33,34,35,36,37]).

**Figure A1:**
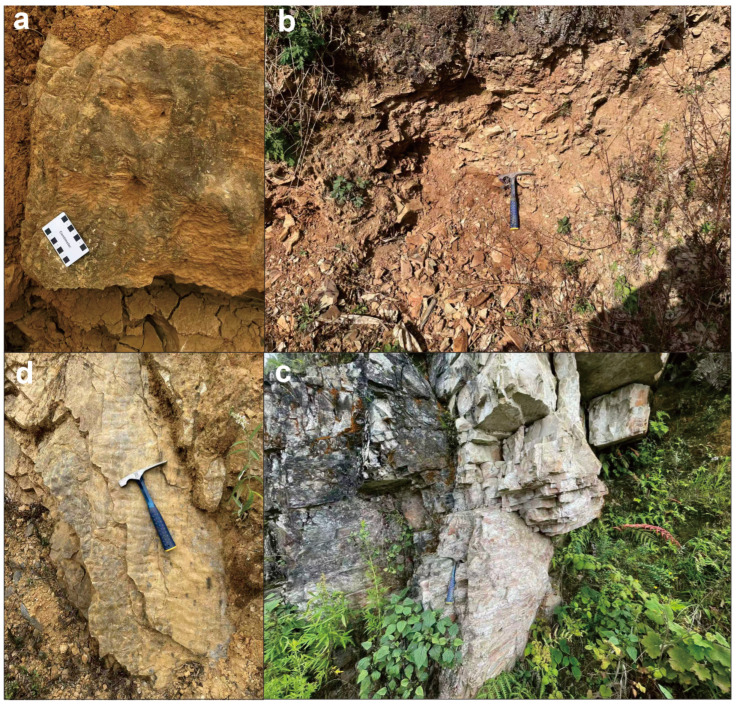
Field photograph showing the stratigraphic outcrop; (**a**) stromatolite from Dayakou Formation; (**b**) field photograph showing the outcrop of argillaceous siltstone of the Tianpeng Formation; (**c**) field photograph showing the outcrop of pinkish-white dolomite of the Longha Formation; (**d**) field photograph showing an outcrop of gray siltstone with ripple marks of the Longha Formation.

The study area lies on the southwestern margin of the South China Block (Appendix A, Figure A1a). The Niuzuodi section in the Bainiuchang area is situated approximately 50 km east of Mengzi City and 30 km west of Wenshan City in southeastern Yunnan Province, where Cambrian and Devonian deposits are well exposed (Figure 1b) [33,34]. The Cambrian stratigraphic sequence in this region comprises, in ascending order, the Dazhai Formation, Dayakou Formation, Tianpeng Formation, and Longha Formation (Figure 1c [33,35,36]). According to previous studies, the Dazhai Formation in the Bainiuchang area consists of oncolitic white dolomitic limestone [33], but it is not exposed at the Niuzuodi section [35]. The Dayakou Formation in this area is composed of stromatolite-rich yellow marl (Appendix A Figure A1a) with local sandstone interbeds [35]. The Tianpeng Formation represents a fossiliferous succession of alternating grayish-yellow, grayish-green, and yellowish-brown argillaceous to silty clastic rocks (Appendix A Figure A1b) and carbonate rocks [33,34,35,36,37]. The Longha Formation features pinkish-white dolomite (Appendix A Figure A1c) at its base, gray siltstone with ripple marks (Appendix A Figure A1d) in its middle and upper parts, and an angular unconformity with the overlying Devonian strata at its top [35]. The lithology and the fossil assemblage from this locality, as documented in previous studies, confirms that the newly collected specimens pertain to the Tianpeng Formation [35,37].

The Tianpeng Formation (Cambrian, Miaolingian Series, Wuliuan Stage) is a shallow-marine platform succession of alternating clastic and carbonate deposits [33,35,38]. The lower member consists predominantly of shale, sandstone, and siltstone with occasional dolomite interbeds, whereas the upper member is characterized by siltstone–dolomite alternations capped by convolute bedding structures (Figure 1c [34,35]). The Tianpeng Formation is in conformable contact with the underlying Dayakou Formation (Cambrian Series 2, Duyunian regional Stage) and the overlying Longha Formation (Miaolingian Series, Guzhangian Stage) (Figure 1c [34]).

Chronostratigraphically, the Tianpeng Formation is younger than the Qiongzhusi Formation (Cambrian Stage 3), which hosts the Chengjiang biota [15], and the Wulongqing Formation (Cambrian Stage 4), which hosts the Guanshan biota, but older than the Longha Formation (Cambrian Guzhangian Stage) [16]. Of these units, the Longha Formation is the only one in southeastern Yunnan from which a BST Lagerstätte—the Fulu biota—has been formally documented to date [39]. No BST-type soft-bodied preservation has previously been reported from the Tianpeng Formation.

The palaeoscolecid specimen described herein was collected from the Niuzuodi section at a stratigraphic level corresponding to 146.4 m (from the base of the formation) of the true thickness of the Tianpeng Formation [35,37]. This horizon lies within the transitional interval between the lower clastic-dominated member and the upper carbonate-rich member (Figure 1c), a position that may be significant for understanding the taphonomic context of the fossil.

## 3. Materials and Methods

### 3.1. Material

**Figure A2:**
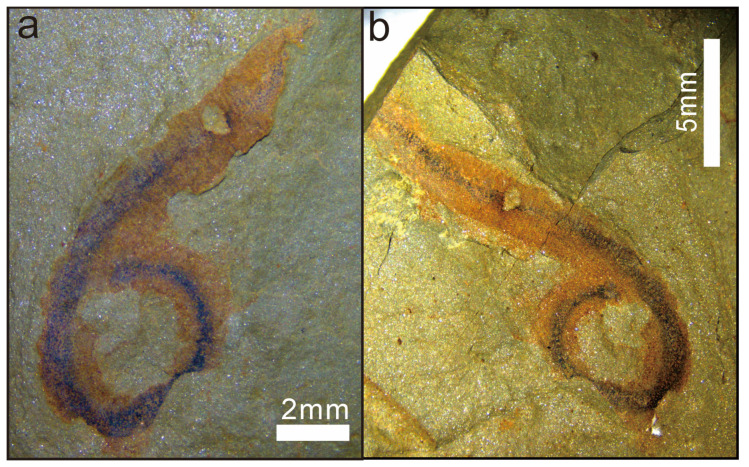
Photomicrograph of the palaeoscolecid specimen; (**a**) photomicrograph of the KUST-GM-F20240701A (impression); (**b**) photomicrograph of the KUST-GM-F20240701B (counterimpression).

The material described herein comprises a single palaeoscolecid specimen (*Wronascolex* sp.) collected from the Tianpeng Formation (Wuliuan Stage) at the Niuzuodi section, Bainiuchang area, southeastern Yunnan (see Section 2 for geological context). The specimen is deposited in the Geological Museum of Kunming University of Science and Technology under catalog number KUST-GM-F20240701A (impression) and KUST-GM-F20240701B (counterimpression); the photograph is shown in Appendix A, Figure A2.

The rock hosting the palaeoscolecid specimen is a grayish-yellow argillaceous siltstone, with a thickness of approximately 30 cm, displaying horizontal lamination at ~1 mm spacing. Hand-specimen and stereo-microscope observations indicate that the rock is composed predominantly of clay minerals and mica, with grain sizes ranging from 0.005 to 0.050 mm (clay to coarse silt). The host rock does not effervesce upon exposure to dilute hydrochloric acid, confirming the absence of carbonate minerals.

**Figure A3:**
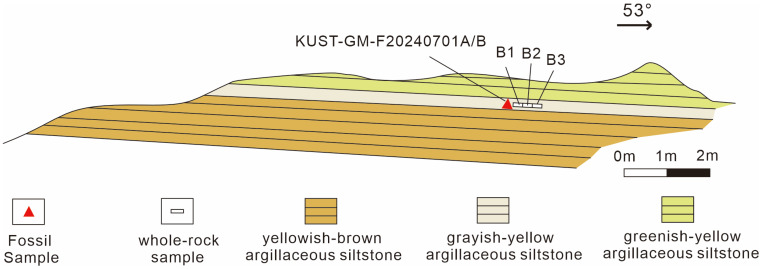
Schematic diagram of the sample location.

The grayish-yellow argillaceous siltstone containing the paleoscolecid specimen is in conformable contact with both the overlying and underlying rocks. These rock layers exhibit significant differences in color, sediment grain size, and fossil species composition. The schematic diagram of the sample location is shown in Appendix A, Figure A3.

**Figure A4:**
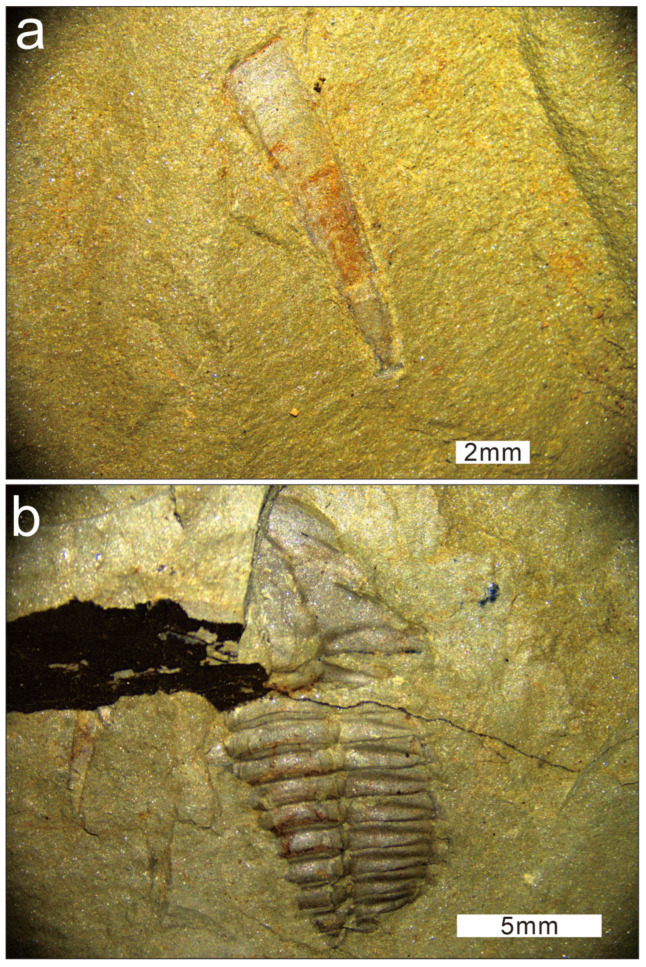
(**a**) Hyolith fossil preserved in yellowish-brown argillaceous siltstone; (**b**) *Kaotaia magna* Lu, 1945 [52] preserved in greenish-yellow argillaceous siltstone.

Beneath the grayish-yellow siltstone containing the worm fossil lies a layer of yellowish-brown argillaceous siltstone, approximately 2.2 m thick, with a bedding thickness of about 2 mm. Stereo-microscope observations indicate that the rock is composed predominantly of clay minerals and mica grain sizes ranging from 0.05 to 0.060 mm. Fossils are rare in this layer, with only a few hyolith fossils identified (Appendix A Figure A4a).

Above the greenish-yellow siltstone containing the worm fossil is another layer of grayish-yellow argillaceous siltstone, 1.5 m thick, with a bedding thickness of approximately 5 mm, and stereo-microscope observations indicate that the rock is composed predominantly of clay minerals and mica grain sizes ranging from 0.015 to 0.085 mm. The mica content in this layer is significantly higher than that in the underlying strata. Hyolith fossils and trilobite fossils are present (Appendix A Figure A4b).

Three additional argillaceous siltstone samples (sample numbers: B1,B2,B3) were collected from the same stratigraphic level (146.4 m from the base of the formation) for whole-rock geochemical analysis, including major elements, trace elements and rare earth elements. Analytical results are presented in Section 6 and Table 1.

### 3.2. Methods

Samples were observed and identified using a Leica S6 (Leica Microsystems, Wetzlar, Germany) stereo-microscope. Optical photomicrographs were captured using a Leica DFC450 (Leica Microsystems, Wetzlar, Germany) microscope camera with LAS v4.8 imaging software. Uncoated specimens were mounted on stubs and analyzed using a Hitachi SU3900 scanning electron microscope (Hitachi, Tokyo, Japan) (SEM) equipped with an Oxford Ultim Max 40 (Oxford Instruments, Abingdon, UK) energy-dispersive X-ray spectrometer (EDS) at the Analytic & Testing Research Center of Kunming University of Science and Technology, operating at an accelerating voltage of 15 kV. All specimens examined in this study are deposited at the Geological Museum of Kunming University of Science and Technology (KUST-GM), Kunming, China.

Whole-rock geochemical analyses were performed at the China Metallurgical Geology Bureau (Shandong Branch) Group Testing Co., Ltd (Jinan, China). The analytical procedures are described below.

Major elements: The flux was a mixture of lithium tetraborate and lithium metaborate, with ammonium nitrate as the oxidizer and a small amount of ammonium bromide as the releasing agent. The sample-to-flux mass ratio was 1:10. The mixture was fused at 1150 °C in a fusion machine to produce glass beads, which were subsequently analyzed using a Thermo Fisher XR-PFX-04U (Thermo Fisher Scientific, Switzerland) X-ray fluorescence spectrometer (XRF). Major and minor element concentrations were determined based on fluorescence intensity. Data reliability was ensured by employing the Chinese granite reference standard GSR-01 as a quality control sample, yielding an analytical precision better than 1% [40].

Trace elements and rare earth elements (REEs): Samples were digested with hydrofluoric acid (HF) and nitric acid (HNO_3_) in sealed Teflon vessels. Hydrofluoric acid was evaporated on a hotplate, followed by further digestion with aqua regia. After appropriate dilution, trace and rare earth element concentrations were determined by external standard calibration using an Agilent 8900 ICP-MS/MS (Agilent Technologies, Santa Clara, CA, USA) triple quadrupole inductively coupled plasma mass spectrometer. Analytical data quality was monitored using the international reference materials AMH-1 and OU-6, with an analytical precision better than 5% [41].

## 4. Systematic Paleontology

Phylum Priapulida Delage et Herouard, 1897 [42];

Class PALAEOSCOLCIDA Conway Morris & Robison,1986 [43];

Order Uncertain;

Family PALAEOSCOLECIFEA Whittard, 1953 [6];

Genus *WRONASCOLEX* Ivantsov et Zhuravlev, 2005 [8].

Genus diagnosis (emended. García-Bellido et al., 2013 [17]): Macroscopic worm with long, slender, cylindrical body covered by annulated cuticle. Each annulation bears one to four rows of *Hadimopanella*-type circular phosphatic sclerites (plates). Plates are round to slightly ovate, with an ornamented upper surface consisting of a single circle of 3–10 peripheral nodes and an occasional central node. Plates are incorporated into a tessellating scleritome of smaller platelets and microplates [10,23].

Type species: *Wronascolex lubovae* Ivanstsov & Wrona, 2004 [12,44];

*Wronascolex* sp.;

*Locality*: Bainiuchang (103°47′ E, 23°29′ N), Southeast Yunnan, South China;

*Horizon*: Sample KUST-GM-F20240701, Tianpeng Formation, Cambrian Miaolingian, Wuliuan Stages;

Material: KUST-GM-F20240701A (impression), KUST-GM-F20240701B (counterimpression).

**Figure 2:**
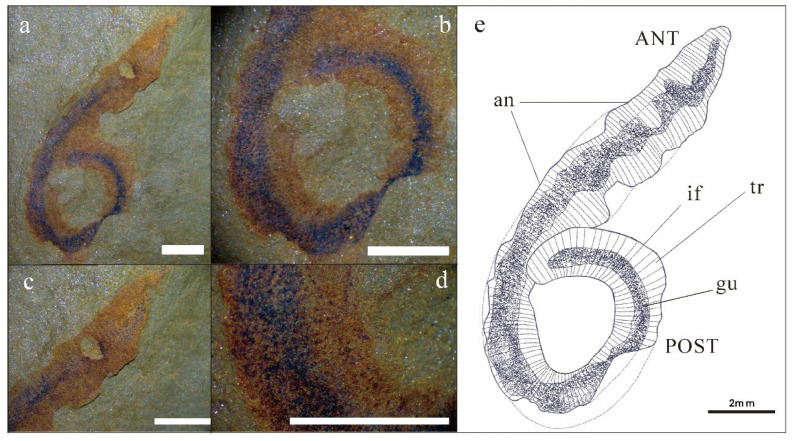
Micrograph and sketch of KUST-GM-F20240701A. All scale bars = 2 mm. (**a**) Micrograph of sample; (**b**) trunk; (**c**) anterior; (**d**) annulations; (**e**) sketch of sample. Abbreviations: ANT, anterior; POST, posterior; an, annulation; gu, gut; tr, trunk; if intersegmental furrow.

Description: This palaeoscolecid specimen is a macroscopic compression fossil preserving a long, cylindrical trunk. The specimen retains a nearly complete trunk with a distinct, dark-colored (black) digestive tract running longitudinally from the anterior to the posterior end. The introvert (proboscis) is not preserved. In plan view, the body is gently arcuate, with the trunk curving slightly to one side (Figure 2a).

A straight digestive tract extends along the entire preserved length of the trunk. The gut is fusiform in outline, attaining a maximum width of 1.5 mm (mean 1.2 mm) in the mid-trunk region and tapering to 0.8 mm posteriorly (Figure 2a), where it terminates in a subtle sac-like expansion (Figure 2b). The preserved (incomplete) trunk measures 34 mm in length, with a body width ranging from 2.1 mm (anterior) to 3.3 mm (posterior).

**Figure 3:**
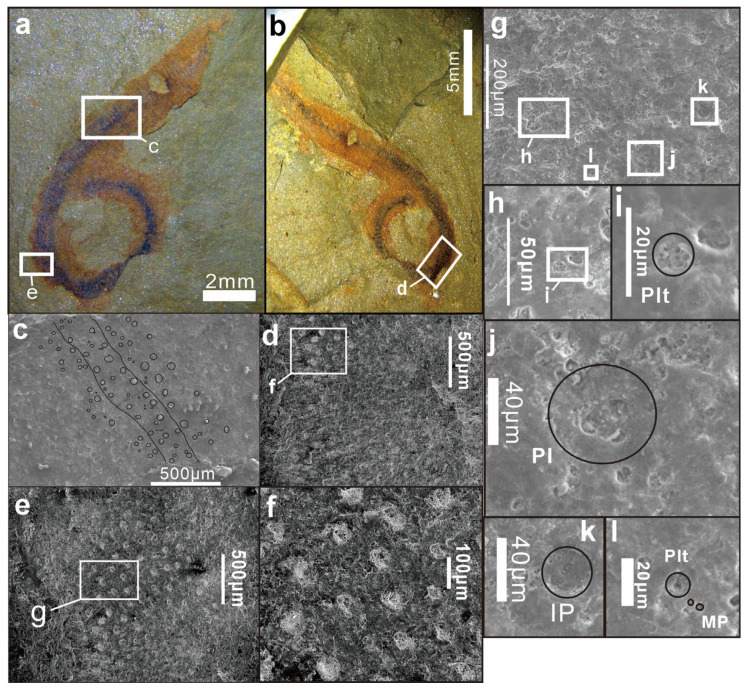
Secondary electron images of the KUST-GM-F20240701A and KUST-GM-F20240701B. (**a**) Micrograph of KUST-GM-F20240701A; (**b**) micrograph of KUST-GM-F20240701B; (**c**) secondary electron image of KUST-GM-F20240701A; (**d**–**f**) secondary electron image of KUST-GM-F20240701B; (**g**,**h**) secondary electron image of KUST-GM-F20240701A, showing the palte, implanted plate and platelet; (**i**) platelet; (**j**) palte; (**k**) implanted plate; (**l**) platelet and microplate. Abbreviations: Pl, plate; IP, implanted plate; Plt, platelet; MP, microplate.

The trunk is densely annulated, although intersegmental furrows are not uniformly distinct, probably owing to compaction and incomplete mineralization (Figure 2c–d). Annulation frequency ranges from five to eight annuli per millimeter. Each annulation bears two nearly transverse rows of *Hadimopanella*-type plates. The plates are sparsely distributed, with no contact between adjacent individuals. Plates are circular to slightly elliptical in outline, with the long axes of elliptical individuals typically aligned parallel to the annulation direction. Plate diameter ranges from 34 to 52 μm. The upper surface of each plate bears 3–7 nodes arranged in a single peripheral circle (Figure 3c–f), occasionally accompanied by a central node.

Between the two primary rows, interstitial (implanted) plates of similar morphology but slightly smaller diameter (26–43 μm) are occasionally present, staggered relative to the primary plates (Figure 3k). In addition to the primary and interstitial plates, numerous platelets are present within the inter-plate spaces of each annulation. Platelets are circular, 6–16 μm in diameter, and bear 1–4 nodes on their upper surface (Figure 3i). Microplates, considerably smaller than the platelets (1–3 μm in diameter), are scattered within the inter-plate spaces of each annulation and bear 1–2 nodes on their surface (Figure 3l).

Remarks: Following García-Bellido et al. [18], the genus *Wronascolex* is diagnosed by the presence of one to four rows of *Hadimopanella*-type sclerites per annulation in macroscopic palaeoscolecid body fossils. Under this taxonomic framework, *Palaeoscolex* Whittard, 1953 [6] is restricted to species bearing *Milaculum*-type sclerites (e.g., the type species *P. piscatorum* Whittard, 1953), whereas all macroscopic palaeoscolecids with *Hadimopanella*-type sclerites refer to *Wronascolex* [11].

The macroscopic morphology and sclerite characteristics of the Bainiuchang specimen (KUST-GM-F20240701A and KUST-GM-F2024070B) are consistent with the diagnosis of *Wronascolex*, particularly in possessing two rows of *Hadimopanella*-type plates per annulation with 3–7 peripheral nodes, together with associated platelets and microplates. However, the specimen differs from all currently described species of *Wronascolex* in plate diameter, node count, and the arrangement of interstitial plates (see Differential Diagnosis above and Comparisons below).

Critically, the introvert (proboscis) is not preserved in this specimen, precluding observation of introvert hooks and scalids—characters that are taxonomically significant at the species level in palaeoscolecids. The absence of these diagnostic features, together with the availability of only a single specimen, precludes definitive species-level assignment. The material is therefore designated as *Wronascolex* sp., pending the discovery of additional, more completely preserved specimens.

The type species of the genus, *Wronascolex lubovae* Ivantsov and Wrona, 2004 [44], was originally described from the Sinsk Formation (Cambrian Series 2, Stage 4) of Siberia [12]. This species is characterized by two rows of *Hadimopanella*-type plates and two rows of platelets per annulation [44]. The plates of *W. lubovae* measure 40–80 μm in diameter and typically bear 5–8 nodes arranged in a peripheral circle, occasionally accompanied by a central node [44]. The platelets measure 30–40 μm in diameter [12]. Notably, microplates have not been reported in *W. lubovae* [12].

In contrast, *Wronascolex* sp. differs from *W. lubovae* in the following respects: (1) a sparser arrangement of plates; (2) smaller plate diameters (34–52 μm vs. 40–80 μm in *W. lubovae*); (3) fewer nodes per plate (3–7 vs. 5–8) (Figure 3j); and (4) the presence of interstitial (implanted) plates between the two primary rows. The term ‘implanted plates’ (sensu Yang et al. [1]) refers to subsidiary plates that develop between the two primary rows of *Hadimopanella*-type plates within each annulation; these are morphologically similar to the primary plates but are characteristically smaller in diameter and more sparsely distributed [1,4]. Implanted plates have not been reported in *W. lubovae* [12,44] but are present in *Wronascolex* sp.

Furthermore, the platelets of *Wronascolex* sp. are considerably smaller (6–16 μm; Figure 3i) than those of *W. lubovae* (30–40 μm). The presence of microplates (1–3 μm in diameter; Figure 3l) in *Wronascolex* sp. constitutes an additional distinction, as microplates have not been reported in *W. lubovae* [8,32].

Following the emendation of *Wronascolex spinosus* Ivanstsov & Wrona, 2004 by Yang et al. [1,44], we present a comparative analysis between *Wronascolex* sp. and the revised diagnosis of *W. spinosus.* Yang et al., 2016 [1] documented the presence of implanted plates in *W. spinosus* specimens from the Shibei Formation (Cambrian Series 2, Stage 4), whose arrangement and dimensions closely resemble those observed in the newly discovered *Wronascolex* sp.

In comparison, *W. spinosus* is characterized by the following features: (1) 1–2 rows of circular to elliptical *Hadimopanella*-type sclerites per annulation [44]; (2) sparsely arranged sclerites, with individual plates measuring 50–60 μm in diameter; and (3) each plate bearing a circle of 4–10 marginal nodes.

While *Wronascolex* sp. closely resembles *W. spinosus* in both sclerite arrangement and plate morphology, it differs in two key aspects: (1) the plates of *Wronascolex* sp. are slightly smaller in diameter, and (2) the number of nodes per plate is generally reduced (fewer than the 4–10 nodes observed in *W. spinosus* [1,12,44]).

However, the most significant distinction lies in the absence of platelets or other sclerite morphotypes in *W. spinosus* [1,12], which are present in *Wronascolex* sp.

In comparison to *Wronascolex* sp., *Wronascolex jianhensis* Yang, 2016 exhibits the following features: (1) two rows of circular to elliptical *Hadimopanella*-type sclerites per annulation [1]; (2) sparsely arranged sclerites, with individual plates measuring 80–90 μm in diameter; and (3) each plate bearing a marginal circle of 2–5 nodes.

While *Wronascolex* sp. shares a similar sclerite arrangement pattern with *W. jianhensis* [1], three critical morphological divergences are observed: (1) plate dimensions: the sclerite plates of *Wronascolex* sp. are significantly smaller in diameter; (2) node count: the plates of *Wronascolex* sp. consistently display a higher number of nodes per marginal circle; and (3) sclerite polymorphism: *W. jianhensis* [1] lacks platelet structures or supplementary sclerite morphotypes, whereas *Wronascolex* sp. exhibits distinct heteromorphism in its sclerite assemblage.

In comparison to *Wronascolex* sp., *Wronascolex yichangensis* Yang, 2016 exhibits the following features: (1) two rows of circular to elliptical *Hadimopanella*-type sclerites per annulation [1]; (2) sparsely arranged sclerites, with individual plates measuring 37–62 μm in diameter; and (3) each plate bearing a marginal circle of 4–7 nodes [1].

While *Wronascolex* sp. closely resembles *W. yichangensis* in sclerite arrangement, plate morphology, and size range, key distinctions are observed in the following aspects: (1) annulation density: *W. yichangensis* exhibits significantly denser annulations (8–13 annulations per mm) than *Wronascolex* sp. (5–8 annulations per mm); and (2) sclerite polymorphism: *W. yichangensis* [1] lacks platelet structures or auxiliary sclerite morphotypes, whereas *Wronascolex* sp. displays distinct heteromorphic sclerite assemblages. These combined morphological divergences suggest taxonomic separation between the two forms [1].

In comparison to *Wronascolex* sp., *Wronascolex iacoborum* García-Bellido et al., 2013 [11] is characterized by the following features: (1) annulation density: sparser annulation patterns (≤5 annulations per mm) compared with *Wronascolex* sp.; (2) sclerite arrangement: two rows of circular to elliptical *Hadimopanella*-type sclerites per annulation, with sclerites densely packed within annulations (in contrast to the looser arrangement in *Wronascolex* sp.); (3) plate morphology: individual plates measure 40–50 μm in diameter, each bearing a uniform marginal ring of 5 nodes, with no central nodes observed; (4) accessory sclerites: possesses platelets (25 μm in diameter) and microplates (15 μm in diameter), both larger than those of *Wronascolex* sp.; and (5) implanted plates: notably absent in *W. iacoborum*, whereas these structures are diagnostic of *Wronascolex* sp. [11].

While *Wronascolex* sp. shares a similar sclerite row configuration and basic plate shape with *W. iacoborum*, the following key distinctions are observed: differences in annulation density, sclerite packing intensity, node architecture, and sclerite polymorphism. These combined morphological divergences provide definitive criteria for taxonomic separation.

**Figure 4:**
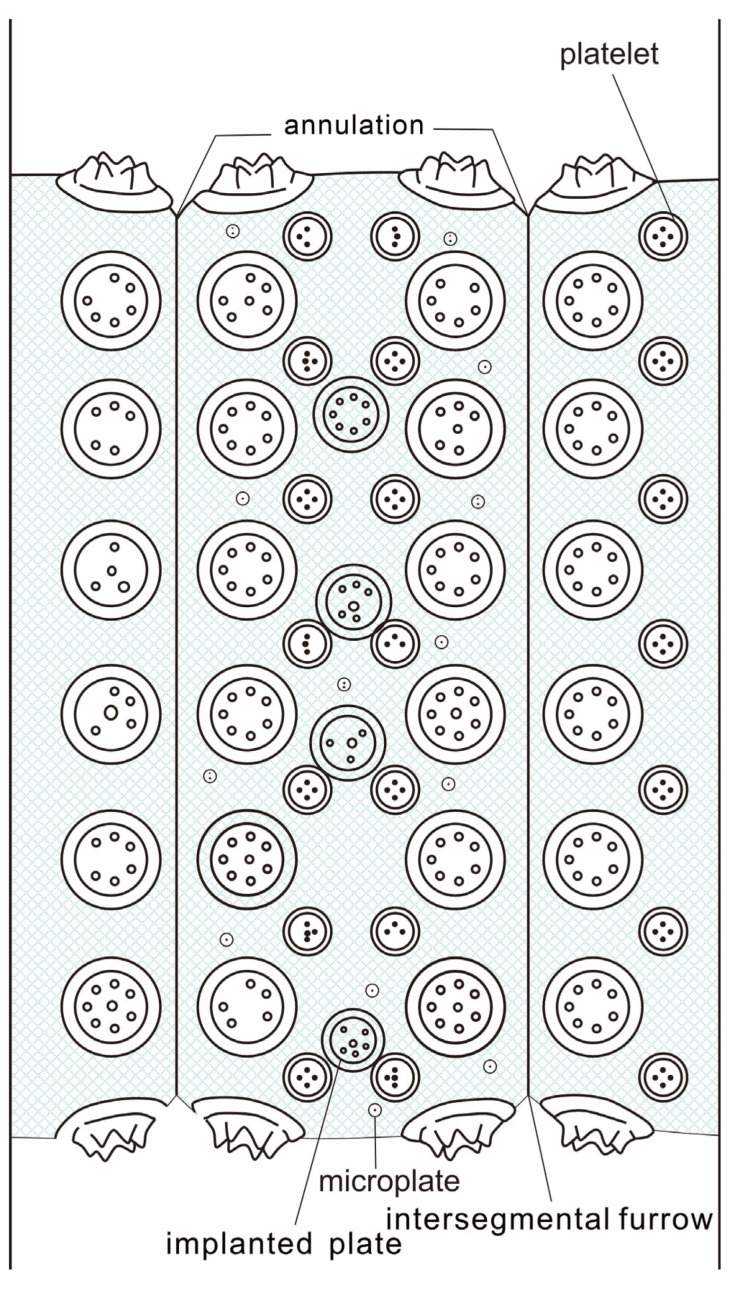
Scleritome of *Wronascolex* sp.

However, the classification scheme proposed by [11] does not provide a comprehensive weighting for the significance of platelets, microplates [11], and other structures in taxonomic differentiation. Therefore, it remains uncertain whether the differences in platelets and microplates (Figure 4) between *Wronascolex* sp. and other species of the genus represent distinctions at the species or genus level. Further studies that incorporate a more detailed evaluation of these morphological features are necessary to clarify their taxonomic implications.

## 5. Characteristics of Fossil Preservation

**Figure 5:**
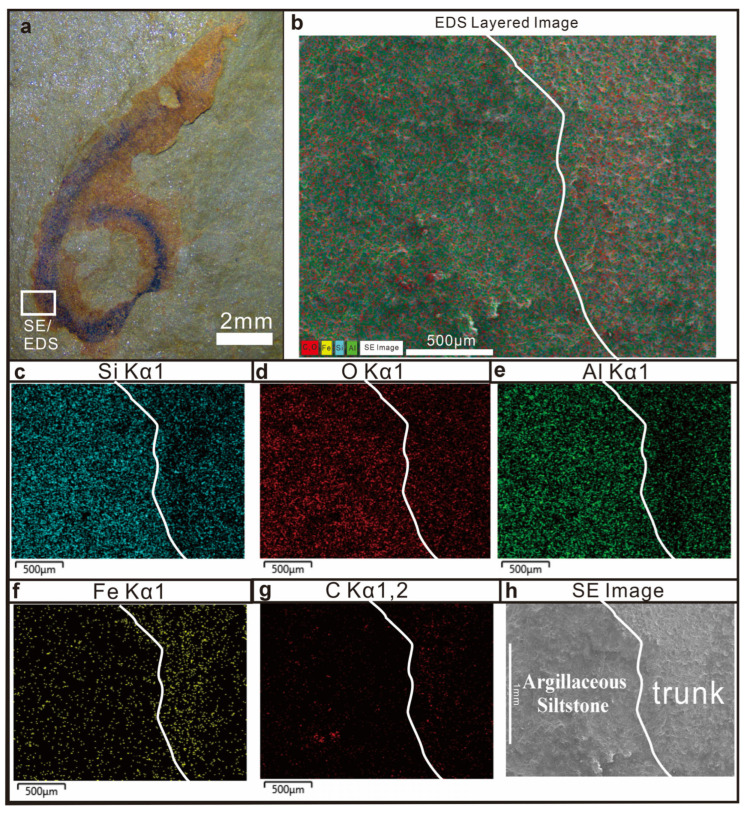
Secondary electron image and SEM-EDS element mapping (Si, O, Al, Fe, C) of KUST-GM-F20240701A (impression). (**a**) Test area; (**b**) EDS layer image of KUST-GM-F20240701A; (**c**) the fossilized tissue is depleted in Si relative to the host rock; (**d**): the fossilized tissue is depleted in O relative to the host rock; (**e**): the fossilized tissue is depleted in Al relative to the host rock; (**f**): the fossilized tissue is significantly enriched in Fe relative to the host rock; (**g**) the tested area shows relatively low carbon content, with the fossilized tissues exhibiting a somewhat higher carbon signal compared to the host rock. (**h**) Secondary electron image of KUST-GM-F20240701A.

**Figure 6:**
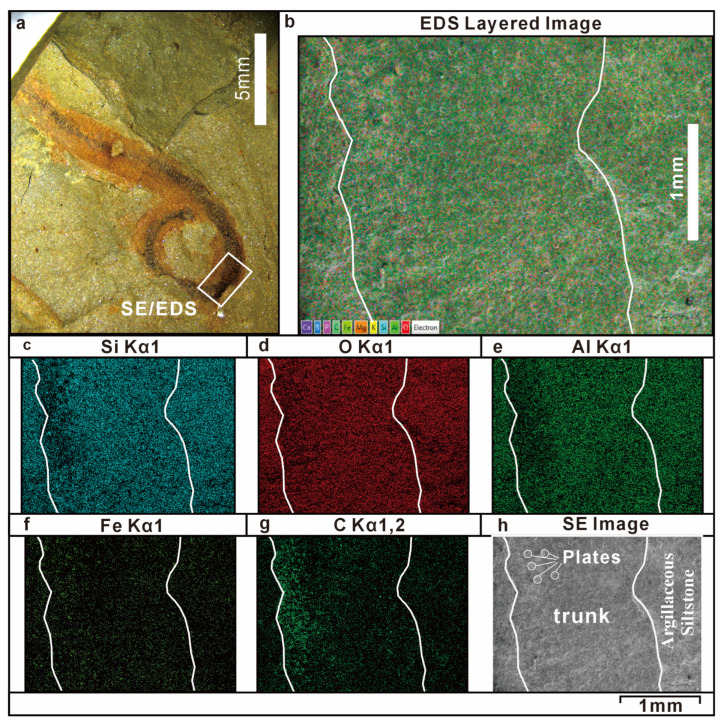
Secondary electron image and SEM-EDS element mapping (Si, O, Al, Fe, C) of KUST-GM-F20240701B (counterimpression). (**a**) Test area; (**b**) EDS layer image of KUST-GM-F20240701B; (**c**) the fossilized tissue is depleted in Si relative to the host rock, particularly in areas where sclerites are distributed; (**d**) the fossilized tissue is depleted in O relative to the host rock; (**e**) the fossilized tissue is depleted in Al relative to the host rock, particularly in areas where sclerites are distributed; (**f**) the fossilized tissue is slightly enriched in Fe relative to the host rock; (**g**): the fossilized tissue is significantly enriched in C relative to the host rock; (**h**) secondary electron image of KUST-GM-F20240701B.

SEM-EDS elemental mapping was performed on the fossil and the adjacent host rock to characterize the taphonomic mode of preservation. The results suggest that the host rock matrix is mainly composed of aluminosilicate minerals, whereas the fossilized tissues appear to show relatively higher concentrations of carbon (C) and iron (Fe). Comparative analysis of the fossilized areas and the surrounding rock matrix indicates differences in the relative concentrations of Si, O, Al, C, and Fe. Specifically, the fossilized tissues were relatively depleted in Si, O, and Al but show relatively higher concentrations of C and Fe relative to the surrounding matrix (Figure 5 and Figure 6). It should be noted that these observations are based on qualitative elemental mapping; the relative differences described here reflect spatial contrasts visible in the EDS maps but have not been confirmed by quantitative compositional analysis.

Within the body of *Wronascolex* sp., the trunk integument is enriched in Fe and C, whereas the gut region shows relatively higher concentrations of Al, Si, and O (Figure 5). The enrichment of Al, Si, and O in the gut may reflect sediment infilling of the digestive tract or in situ formation of aluminosilicate (clay) minerals during early diagenesis, a phenomenon documented in other BST deposits [15,20]. The Fe and C enrichment of the trunk integument is consistent with a taphonomic mode involving carbonaceous compression accompanied by iron mineralization—features commonly associated with Burgess Shale-type (BST) preservation [15].

It should be noted, however, that the speciation of iron within the fossil remains unresolved on the basis of SEM-EDS data alone. The elevated Fe concentrations could reflect the presence of iron oxides, iron oxyhydroxides, iron-bearing clay minerals, or pyrite (FeS_2_). Pyritization of soft tissues is a well-documented auxiliary taphonomic pathway in several BST deposits, and the potential role of early diagenetic pyritization in the preservation of *Wronascolex* sp. cannot be excluded. Future work employing X-ray diffraction (XRD) to determine the iron mineral phases, and micro-computed tomography (micro-CT) to assess possible three-dimensional preservation of fine anatomical structures, would provide critical constraints on the taphonomic pathway(s) involved. These taphonomic features are consistent with those reported from other BST deposits globally, and suggest that the Bainiuchang locality may preserve fossils through a broadly similar combination of organic stabilization and authigenic mineralization, although further analytical work is needed to confirm this interpretation.

The Bainiuchang *Wronascolex* sp. thus provides new data on the taphonomy of palaeoscolecid body fossils and adds to the growing body of evidence for BST-style preservation in the Miaolingian of South China.

## 6. Geochemical Data

For this study, three argillaceous siltstone samples from the same horizon as the fossil specimens were collected and analyzed for their whole-rock major, trace, and rare earth element (REE) compositions (Table 1). Based on these geochemical data, geochemical redox proxies were employed to assess the redox conditions during fossil preservation.

**Table 1:** Major (%), trace (10^−6^) and rare earth (10^−6^) contents of argillaceous siltstone in fossil locality.

Compositions	K_2_O	Al_2_O_3_	CaO	TFe_2_O_3_	MgO	Na_2_O	SiO_2_	MnO	P_2_O_5_	TiO_2_	V	Co	Ni	Cu	Zn
	%	%	%	%	%	%	%	%	%	%	10^−6^	10^−6^	10^−6^	10^−6^	10^−6^
B1	5.74	19.94	0.26	5.74	1.89	0.17	59.01	0.02	0.11	0.83	126.00	13.60	47.10	40.20	82.80
B2	4.15	13.67	0.11	4.55	1.42	0.17	70.78	0.03	0.12	0.76	87.70	11.40	34.90	17.80	50.40
B3	4.71	13.31	0.23	4.42	1.85	0.15	69.67	0.03	0.17	0.74	105.00	12.70	29.50	12.70	57.80
compositions	La	Ce	Pr	Nd	Sm	Eu	Gd	Tb	Dy	Ho	Er	Tm	Yb	Lu	Y
	10^−6^	10^−6^	10^−6^	10^−6^	10^−6^	10^−6^	10^−6^	10^−6^	10^−6^	10^−6^	10^−6^	10^−6^	10^−6^	10^−6^	10^−6^
B1	57.80	91.30	11.80	41.10	6.31	1.11	5.11	0.81	4.57	0.90	2.75	0.42	2.68	0.41	23.50
B2	48.60	93.90	11.20	41.60	7.64	1.39	6.66	1.05	5.84	1.13	3.38	0.52	3.30	0.53	27.30
B3	64.90	126.00	14.90	55.50	10.20	1.81	9.07	1.37	7.29	1.40	4.15	0.63	3.86	0.62	37.50

Note: TFe_2_O_3_ represents the total iron content in a sample, calculated and expressed as Fe_2_O_3_, regardless of the original forms of iron present (e.g., FeO, Fe_2_O_3_, or other iron-bearing minerals).

### 6.1. Analytical Methods and Proxy Selection

Three geochemical redox indexes widely applied to clastic rocks were utilized, including Cu/Zn, Ce/La, and cerium anomaly normalization (Ce_anom_).

The Cu/Zn ratio reflects differences in oxygen fugacity during deposition, as Cu and Zn develop distinct sedimentary zoning patterns under varying redox conditions [45]. Cerium (Ce) is a redox-sensitive rare earth element, existing in both +3 and +4 valence states; variations in redox conditions produce Ce anomalies that serve as tracers for palaeo-marine redox environments. To enhance the reliability of the interpretation, both the Ce/La ratio [46] and the Ce_anom_ [47] were applied. Ce_anom_ is calculated using the formula:

Ce_anom_ = log[3Ce_N_/(2La_N_ + Pr_N_)]

REE data are normalized to the Upper Continental Crust (UCC) using the values from Taylor and McLennan (1985) [48].

The thresholds used for interpretation are summarized as follows:

(1) Cu/Zn: >0.63 oxidizing; 0.63–0.5 weakly oxidizing; 0.5–0.38 transitional; 0.38–0.21 weakly reducing; <0.21 strongly reducing [45];
(2) Ce/La: ≥2 reducing; 1.8–2 weakly reducing; 1.5–1.8 suboxic; <1.5 oxic [46];
(3) Ce_anom_: >−0.1 anoxic; <−0.1 oxic [48].

### 6.2. Analytical Results

The calculated redox indices are presented in Table 2. The Cu/Zn ratios range from 0.22 to 0.49. The Ce/La values range from 1.58 to 1.94. The Ce_anom_ values range from −0.10 to −0.03.

### 6.3. Interpretation

The Cu/Zn ratios (0.22–0.49) indicate a depositional environment transitional between weakly reducing and intermediate redox conditions. The Ce/La values (1.58–1.94) suggest deposition under suboxic to weakly reducing conditions. The Ceanom values (−0.10 to −0.03) indicate that the fossil-bearing clastic rocks were deposited across a redox gradient near the anoxic–oxic boundary. Taken together, these proxies suggest that the fossil-forming depositional environment represents a redox transition from anoxic to oxic conditions. Such a redox gradient is broadly similar to that associated with the Burgess Shale-type Lagerstätte discovered by Kimmig and Pratt in the Mackenzie Mountains, Canada [49]. Kimmig and Pratt’s research demonstrates that the Ravens Throat River Lagerstätte was deposited under a predominantly oxic water column, with preservation facilitated by specific sediment composition and clay mineral interactions rather than persistent anoxia—conditions that, together with the oxic–anoxic gradient near the sediment–water interface, are particularly favorable for the preservation of soft-bodied fossils [49].

## 7. Discussion

Paleoscolecid worm fossils are rare in southeastern Yunnan. The *Wronascolex* sp. specimen recovered from the Tianpeng Formation represents the oldest known occurrence of this group in the region, predating the Fulu Biota documented by Peng and Yang [38]. This potential Konservat-Lagerstätte postdates the Chengjiang and Guanshan biotas (Early Cambrian, eastern Yunnan). Conversely, it predates the Fulu biota, the only confirmed Burgess Shale-type Lagerstätte in southeastern Yunnan to date. Thus, the discovery of this *Wronascolex* sp. establishes a new temporal benchmark for understanding paleontological evolution in this area [39].

The preservation of *Wronascolex* sp. in this study—characterized by relatively higher concentrations of carbon and iron in the fossilized areas compared to the surrounding rock matrix—shows some features that are broadly reminiscent of Burgess Shale-type fossil preservation. The fossils display complete alignment along bedding planes, suggesting deposition under low-energy hydrodynamic conditions. Preservation in several coeval BST Lagerstätten, such as the Guanshan [16], Kaili [25], and Balang biotas [50], is predominantly characterized by carbonaceous films and pyritization. Energy-dispersive X-ray spectroscopy (EDS) mapping of *Wronascolex* sp. revealed enrichment of iron and carbon in the trunk region; however, EDS analysis further demonstrated that iron occurs mainly in oxidized phases rather than as pyrite. This interpretation is further supported by redox proxy analyses of the host rock. This redox anomaly implies possible post-burial iron diagenesis, although conclusive evidence for oxidative alteration requires additional geochemical investigation.

Therefore, we suggest that the Bainiuchang area warrants further investigation as a possible locality for Burgess Shale-type preservation. Future work would benefit from expanded fossil excavation and research in the region. However, because Bainiuchang lies within an active superlarge silver polymetallic mining district, ongoing mining operations may present logistical challenges. To mitigate these, potential approaches could include integrating additional remote sensing data to identify alternative sections, and seeking international collaboration to enhance the global scientific profile of the area and promote the protection of this unique fossil locality.

Furthermore, the occurrence of *Wronascolex* sp. bearing Burgess Shale-type features further suggests the possible presence of an undiscovered Burgess Shale-type Lagerstätte within the Tianpeng Formation of southern China. However, the current evidence is insufficient to fully support this conclusion, and future research—including the collection of additional fossil specimens and more detailed sedimentological and taphonomic analyses—will be necessary to substantiate this interpretation.

**Figure 7:**
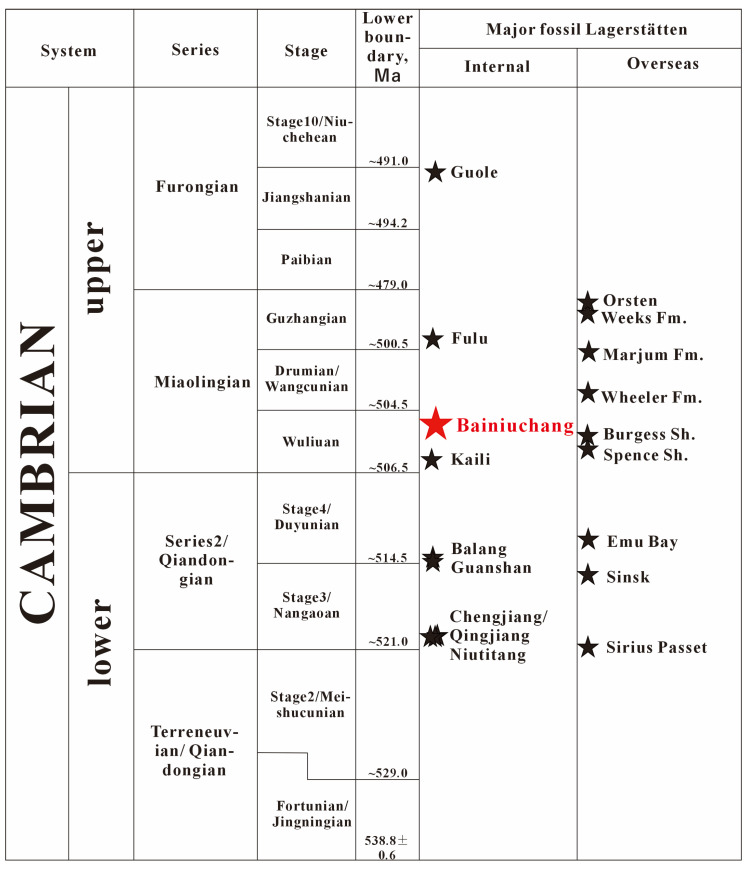
Chart showing global and Chinese chronostratigraphic subdivisions of the Cambrian System, the stratigraphic positions of Cambrian major fossil Lagerstätten (black stars), and the Bainiuchang fossil locality (red star). Abbreviations: Fm., Formation, Sh., Shale. Chart based on data from reference [39]; stratigraphic ages follow the International Chronostratigraphic Chart (v2024-12) [51], data sources: https://stratigraphy.org/chart/, accessed on 6 April 2026.

Finally, from a chronological perspective, the Tianpeng Formation, where *Wronascolex* sp. was discovered in the Bainiuchang area, is stratigraphically younger than the Qiongzhusi and Wulongqing formations that host the Chengjiang and Guanshan biotas in eastern Yunnan, yet older than the Longha Formation that yields the Fulu Biota (Figure 7). If a new Burgess Shale-type Lagerstätte can be established in the Tianpeng Formation through subsequent work, this would provide new insights into our understanding of Cambrian biological evolution and paleoenvironmental changes in Yunnan.

## 8. Conclusions

In this study, we report a new occurrence of the genus *Wronascolex* from the Cambrian (Wuliuan Stage) Tianpeng Formation in the Bainiuchang area of southeastern Yunnan Province, China. The specimen preserves some soft tissues. Although certain diagnostic features of this specimen are distinctive, the absence of several key characters precludes a definitive specific assignment; thus, we currently refer to it as *Wronascolex* sp. Pending the collection of additional material, a formal species description may be possible in the future.

The preservation mode of *Wronascolex* sp. in this study—characterized by carbonaceous compression and ferruginous film coating—exhibits features broadly consistent with Burgess Shale-type fossil preservation. Preliminary redox proxy analyses of the host rock yield values broadly comparable to those reported from some BST deposits, although these data remain limited. Taken together, these observations suggest that the Bainiuchang area warrants further investigation for the possible presence of a Burgess Shale-type Lagerstätte, but this interpretation remains speculative and requires confirmation through additional fossil discoveries and more detailed sedimentological and taphonomic work.

Future work will involve expanded fossil excavation and research in the region. However, because Bainiuchang lies within an active superlarge silver polymetallic mining district, ongoing mining operations may present logistical challenges. To mitigate these, we plan to integrate additional remote sensing data to identify alternative sections, and to seek international collaboration to enhance the global scientific profile of the area and promote the protection of this promising fossil locality.

## Figures and Tables

**Table 2:** Redox indices of argillaceous siltstone in fossil locality.

Samples	Cu/Zn	Ce/La	Ce_anom_
B1	0.49	1.58	−0.10
B2	0.35	1.93	−0.04
B3	0.22	1.94	−0.03

## Data Availability

The original contributions presented in this study are included in the article. Further inquiries can be directed to the corresponding authors. The specimens mentioned in this article have been stored in the Geology Museum of Kunming University of Science and Technology. If you need to inquire about them, please contact the authors.
